# Characteristics of the Proteolytic Enzymes Produced by Lactic Acid Bacteria

**DOI:** 10.3390/molecules26071858

**Published:** 2021-03-25

**Authors:** Marek Kieliszek, Katarzyna Pobiega, Kamil Piwowarek, Anna M. Kot

**Affiliations:** Department of Food Biotechnology and Microbiology, Institute of Food Sciences, Warsaw University of Life Sciences—SGGW, Nowoursynowska 159 C, 02-776 Warsaw, Poland; katarzyna_pobiega@sggw.edu.pl (K.P.); kamil_piwowarek@sggw.edu.pl (K.P.); anna_kot@sggw.edu.pl (A.M.K.)

**Keywords:** proteolytic enzymes, lactic acid bacteria, proteolysis

## Abstract

Over the past several decades, we have observed a very rapid development in the biotechnological use of lactic acid bacteria (LAB) in various branches of the food industry. All such areas of activity of these bacteria are very important and promise enormous economic and industrial successes. LAB are a numerous group of microorganisms that have the ability to ferment sugars into lactic acid and to produce proteolytic enzymes. LAB proteolytic enzymes play an important role in supplying cells with the nitrogen compounds necessary for their growth. Their nutritional requirements in this regard are very high. Lactic acid bacteria require many free amino acids to grow. The available amount of such compounds in the natural environment is usually small, hence the main function of these enzymes is the hydrolysis of proteins to components absorbed by bacterial cells. Enzymes are synthesized inside bacterial cells and are mostly secreted outside the cell. This type of proteinase remains linked to the cell wall structure by covalent bonds. Thanks to advances in enzymology, it is possible to obtain and design new enzymes and their preparations that can be widely used in various biotechnological processes. This article characterizes the proteolytic activity, describes LAB nitrogen metabolism and details the characteristics of the peptide transport system. Potential applications of proteolytic enzymes in many industries are also presented, including the food industry.

## 1. Introduction

The development of global industry and the achievement of a high level of knowledge on the nature of enzymes have allowed enzymes to be used for the production of high-quality consumer goods, thus proving their industrial significance. Enzymes perform an extremely important function; that is, they act as biocatalysts, mediate all anabolic and catabolic pathways, and reduce the activation energy of biochemical reactions [1]. Although the catalytic activity of enzymes has been known to humans for many centuries, recent advances in the global biotechnology industry have enabled a wider use of these biocatalysts. Currently, hydrolytic enzymes are very popular and widely used [2,3].

The lactic acid bacteria group consists of species that are among the best-studied microorganisms. What is more, proteolysis is one of the particular physiological properties of lactic acid bacteria of which important knowledge has been attained. The proteolytic system of lactic acid bacteria is related to the utilization of casein and supplies the cells with essential amino acids during their growth in milk. Lactic acid bacteria and their proteolytic system play a crucial role when it comes to the organoleptic properties of fermented milk products. Except for the processing of nutrients, cellular proteolysis is responsible for controlling the quality of polypeptides and regulatory mechanisms by maintaining an appropriate level of regulatory proteins, and, when necessary, removing them [4]. The proteolytic system of lactic acid bacteria comprises proteinases, peptidases, and specific transport proteins. Proteinases cleave casein into peptides, then peptidases (intracellular) degrade peptides to amino acids and smaller peptides. Transport systems are responsible for transferring amino acids and peptides across the cytoplasmic membrane [5].

The increasing demand for proteolytic enzymes has prompted the need to seek new living organisms that can serve as sources of these enzymes, as well as to perform in-depth analysis of the already available genetic material. Presently, the vast majority of enzymes are produced by recombinant microbial strains, because of which biocatalysts are much cheaper than the proteins isolated from representatives of natural producers [6,7]. Many researchers have focused their interest on understanding the activity, regulation, and physiological function of the proteolytic system of lactic acid bacteria [8,9,10,11].

This manuscript depicts the review of the components of the proteolytic system in lactic acid bacteria that are associated with food fermentations. Special emphasis has been placed on, for example, the structure of extracellular proteinases, the peptide transport system, regulation of the proteolytic system, and industrial use of proteolytic enzymes, including by the dairy, baking and brewing industries.

## 2. Characteristics of Lactic Acid Bacteria

Lactic acid bacteria (LAB) are a group of microbes with increasing importance in biotechnology. They are widely used in milk processing. Traditionally, the use of LAB was mainly limited to the dairy, fermentation, fruit and vegetable, and animal feed industries. The importance of lactic acid bacteria is constantly growing because they are considered safe for humans and animals (Generally Recognized As Safe—GRAS) and show many beneficial effects on human health [12,13,14,15,16].

LAB are a large group of bacteria with the ability to ferment sugars into lactic acid. Lactic acid fermentation is an intracellular, anaerobic enzymatic process of converting sugars to lactic acid, which is one of the approaches to obtain the energy necessary for cell life processes in anaerobic conditions [17,18,19]. Morphologically, lactobacilli are not a homogeneous group. They include rods arranged in chains, such as the genus *Lacticaseibacillus*, *Carnobacterium*, *Weissella*; cocci arranged in chains, such as species of *Lactococcus*, *Enterococcus*, *Vagococcus*; and *Oenococcus* (known as *Leuconostoc oeni*), which was formerly referred to as *Leuconostoc*, and are arranged in the form of tetrads, such as the genus *Tetragenococcus*. These are relatively anaerobic, gram-positive, and nonspore-producing bacteria that usually do not have flagellas [12,13,17,20,21].

LAB can produce proteolytic enzymes inside the cell as well as in the external environment. The optimum pH for the development and growth of proteolytic bacteria in milk is 7–7.5. A low temperature inhibits the growth and development of these bacteria and decreases their enzymatic activity. Temperatures above that are optimal for bacterial growth rapidly inactivate proteolytic enzymes. In some cases, irreversible loss of proteolytic enzyme activity is observed after prolonged storage at low temperatures [22,23,24]. Many proteolytic bacteria have psychrotrophic properties. It should be noted that these bacteria can degrade milk proteins. This aspect has been observed in both bacilli and lactic streptococci, although to a lesser extent than that in typical proteolytic bacteria such as *Proteus*, *Pseudomonas*, *Alcaligenes*, *Acinetobacter*, *Bacillus*, *Clostridium*, and some strains of *Micrococcus* [25].

LAB can degrade proteins, as exemplified by casein. The final products of this decomposition are most often the amino acids used by LAB as a source of nitrogen. Thermophilic lactobacilli species have stronger proteolytic abilities than rods or streptococci; however, each species has strains with highly varying activity in this respect. Depending on the species, subspecies, and even the strain, LAB show very diverse proteolytic activity. Among the cocci, the most active species are *Lactococcus salivarius* subsp. *thermophilus* (formerly named *Streptococcus salivarius* subsp. *thermophilus*) and *Lc. lactis* subsp. *cremonis*, while the least active is *Lc. lactis* subsp. *lactis*. Of the lactobacilli used in the dairy industry, the most proteolytically active species are *Lacticaseibacillus casei*, *Lactobacillus delbrueckii* subsp. *bulgaricus*, *Lb. helveticus*, and *Lb. acidophilus*, while the least active species is *Lactiplantibacillus plantarum*. In general, the activity of rods is greater than that of lactic streptococci [24,26,27,28,29].

When discussing the proteolytic enzymes of LAB, it is worth mentioning lactococcal strains. Proteolysis of casein forms so-called bitter peptides, which are the cause of the bitter taste of dairy products [30].

## 3. Enzymatic Activities of Proteases

**Figure 1:**
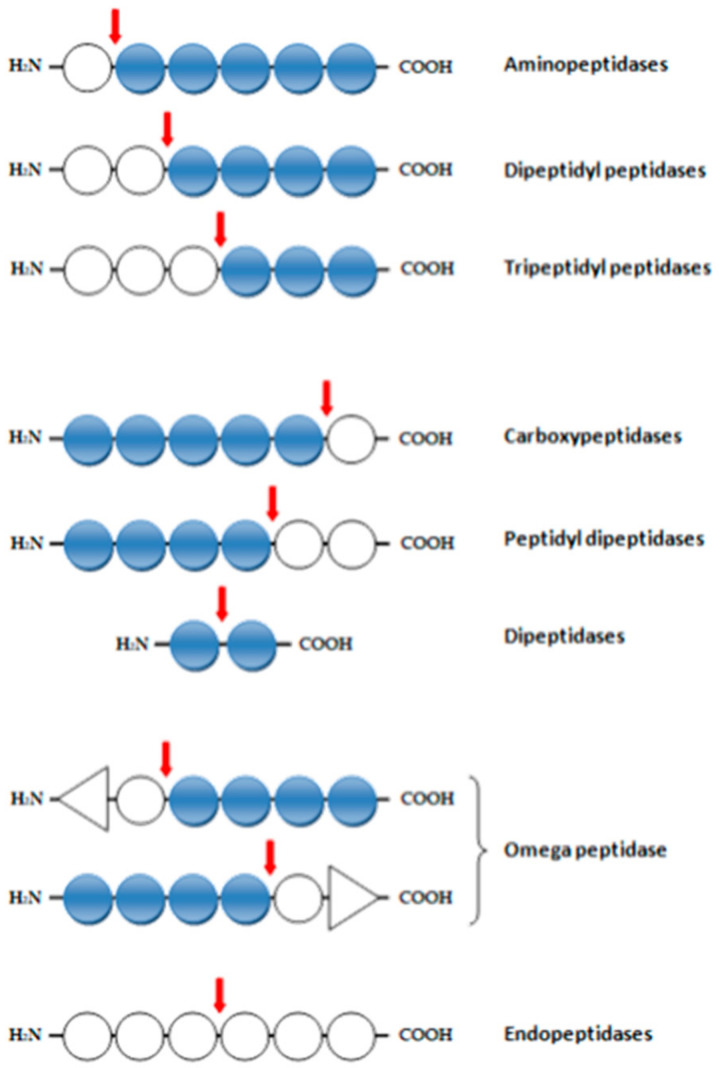
Classification of the activity of proteolytic enzymes. Arrows indicate the site of action of the enzyme. Triangles indicate blocked ends of the polypeptide chain. The white dots are the amino acid residues in the polypeptide chain. The blue dots are terminal amino acids.

Proteolytic enzymes can be classified as exopeptidases or endopeptidases (proteinases) (Figure 1). The first group cleave the peptide bond proximal to the amino or carboxy termini of the substrate. Endopeptidases cleave peptide bonds distant from the termini of the substrate. On the basis of their chemical mechanism of catalysis of the hydrolysis of amide bonds in peptide substrates, proteolytic enzymes can be divided into following groups: serine proteinases (EC 3.4.21), cysteine proteinases (EC 3.4.22), aspaspartyl proteinases (EC 3.4.23), metalloproteinases (EC 3.4.24), and threonine peptidases (EC 3.4.25) [31,32,33].

Additionally, there is the glutamic protease group, which is synthetized only by filamentous fungi [34].

The exopeptidases (EC 3.4.11–EC 3.4.19) include the following groups:

- aminopeptidases (EC 3.4.11), dipeptidyl peptidases (EC 3.4.14), and tripeptide peptidases (EC 3.4.14), which cleave one, two, or three amino acid residues from the N-terminus;
- Ep tripeptidases (EC 3.4.11), – which remove a terminal amino acid from a tripeptide;
- carboxypeptidases (EC 3.4.12): serine (EC 3.4.16), metallocarboxypeptidase (EC 3.4.17), and cysteine (EC 3.4.18), which remove one amino acid residue from the C-terminus;
- dipeptidases (EC 3.4.13), which hydrolyze the peptide bond in the dipeptide;
- peptidyl dipeptidases (EC 3.4.15), which remove two residues from the C-terminus,
- omega peptidases (EC 3.4.19), which remove terminal residues that have neither a free α-amino nor α-carboxyl group [35,36,37,38].

## 4. Nitrogen Metabolism of Bacteria—Proteolysis

LAB have a rapid intracellular metabolism. They require certain energy sources for growth and development. They primarily use carbohydrates and proteins. These bacteria require many free amino acids for their development. For example, *Lactococcus* requires glutamic acid, glycine, leucine, isoleucine, histidine, methionine, and valine to grow properly [39,40,41]. The amount of free amino acids and nitrogen components in milk in a form directly absorbed by bacteria for lactic acid fermentation is insufficient to meet all needs; hence, these bacterial species must obtain these ingredients by decomposing milk proteins, mainly casein [42].

Proteinases of LAB are generally different-molecular-weight proteins. They are synthesized in bacterial cells as preproenzymes. In lactococci, *prtP* genes can either be plasmid- or genome-encoded, whereas CEPs synthesized from lactobacilli are genome-encoded. Despite the high structural similarity, lactic acid bacterial proteins show differences in substrate specificity (Table 1). They show a very strong preference for hydrophobic parts of casein. In the first stage of proteolysis, extracellular proteolytic enzymes (CEPs) degrade casein milk protein to smaller sizes. Casein, as the main milk protein, constitutes approximately 80% of all milk proteins, and can be classified into four fractions: αs1-, αs2-, β-, and κ-casein, each containing a very large number of proline residues; this prevents the formation of *α*-helix and *β* structure of polypeptide chains and promotes the formation of random scrolls. Consequently, these secondary structural features lead to the formation of an unfilled, open molecule that is susceptible to CEP [4].

Proteinases are the first enzymes involved in the hydrolysis of casein [43]. *Lc. lactis* has two extracellular enzymes: lactocepin I (PI-) and lactocepin III (PIII-) [44]; these enzymes are classified according to their specificity for individual fractions of αS1-, β- and κ-casein [45]. Lactocepin I (PI-) mainly attacks β-casein with a highly hydrophobic characteristic and high proline content mainly in the C-terminal fragment of the polypeptide chain. β-Casein is degraded to more than 100 different oligopeptides composed of 4 to 30 amino acid residues, but most peptides contain 4 to 10 amino acid residues [46]. Juillard et al. [47] showed that the bonds most susceptible to this enzyme are those formed by glutamine and serine residues at the C-terminus of the peptide chain over a stretch of 60–105 amino acids. κ-Casein is cleaved to smaller sizes by PI and PIII [46]. PIII can degrade αs1-, β- and κ-casein equally well [48]. Thus, proteinases belonging to PIII can hydrolyze a greater number of peptide bonds than type I proteinases.

Peptides formed due to casein degradation by proteinases bound to the cell wall of LAB (PrtP) are transported into the cells through the functioning of the Opp, Dpp, and DtpT systems. Their further degradation is carried out by endopeptidases (oligopeptidases) and aminopeptidases. Endopeptidases cleave intracellular bonds in peptides, while aminopeptidases remove amino acids from the N- and C-termini of peptides. These enzymes represent metalloenzymes, but only a small number of them belong to the group of cysteine and serine peptidases. A large number of these enzymes have been isolated and biochemically characterized from *Lc. thermophilus* and various strains of *Lactococcus* and *Lacticaseibacillus*. Aminopeptidases (PepN and PepC) and X-prolyl dipeptidyl aminopeptidase (PepX) are the first enzymes that act on oligopeptides. A common feature of endopeptidases is their inability to hydrolyze native casein fractions, but they can hydrolyze the internal bonds of casein derivatives. For example, αs1-casein fragment (1–23) and β-casein fragment (193–209) are the most preferred substrates for LAB endopeptidases. Recently, unique cleavage specificity has been demonstrated in αs1-casein fragment (1–23) and in proline residues of β-casein fragment (203–209), for PepO from the nonstandard bacterial strain *Lacticaseibacillus rhamnosus* HN001 and for PepO2 from the starter bacterial strain *Lb. helveticus* CNRZ32 [4].

**Table 1:** Selected proteolytic enzymes.

Enzymes.	Microorganism	Substrate	Reference
PrtP	*Lacticaseibacillus rhamnosus* CGMCC11055	β-casein	[49]
-	*Limosilactobacillus fermentum* R6	casein	[8]
PrtR	*Lacticaseibacillus rhamnosus* OXY	azocasein	[29]
PrtP	*Lacticaseibacillus paracasei* BL312(VSL#3)	casein	[50]
-	*Lacticaseibacillus casei* PRA205	β-, αs1-, κ-, αs2-caseins	[51]
PrtP	*Lactiplantibacillus plantarum* BGSJ3–18	β-casein	[52]
PrtS	*Streptococcus thermophilus* 4F44	β-casein	[53]
PrtS	*Streptococcus thermophilus*	β-, αs1- and αs2-CN	[54]
PrtB	*Lactobacillus delbrueckii* subsp. *bulgaricus* 92059	β-, α-casein	[55]
PrtL	*Lactobacillus delbrueckii* subsp. *lactis* CRL 581	αs1-, β-, and κ-casein	[56]
PrtH	*Lactobacillus helveticus* CNRZ32	casein	[57]
PrtR	*Lacticaseibacillus rhamnosus* BGT10	β-casein	[58]

Products formed from the action of oligopeptidases and amidopeptidases are substrates for di- and tripeptidases. Their enzymatic activity in individual strains is varied. Because of hydrolysis of oligopeptides by peptidases, mainly aminopeptidases and PepX, the resulting di/tripeptides are susceptible to additional cleavage by tripeptidases PepT and dipeptidases PepV and PepD. These enzymes prefer peptides containing hydrophobic amino acids such as leucine, methionine, phenylalanine, or glycine. Other peptidases characterized by greater substrate specificity include PepA, which releases a three- to nine-amino-acid residue from the N-terminus of the peptide chain. PepP prefers tripeptides with proline in the middle position. PepR and PepI interact with proline-containing dipeptides in the penultimate position, PepQ, cleaving dipeptides with proline in the second place, and PepS, showing preference for peptides containing two to five residues with an Arg residue or an aromatic amino acid at the N terminal [4,45].

The last hydrolysis products are free amino acids, which are a rich source of nitrogen. Enzymatic breakdown of proteins is particularly important for LAB, because it provides them with the free amino acids needed for the synthesis of their own proteins and growth, and allows a significant reduction in energy expenditure on de novo amino acid synthesis.

## 5. Structure of Extracellular Proteinases

**Figure 2:**
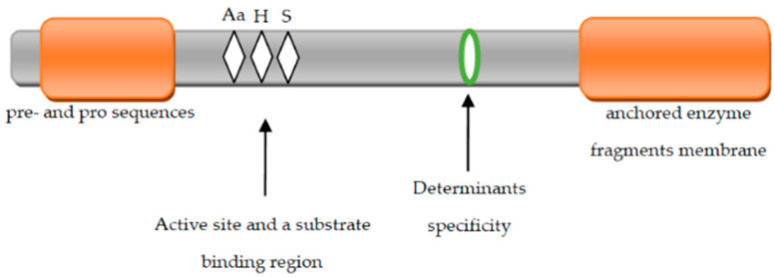
Functional regions in the structure of proteinases associated with the cell wall of LAB. Aa, H, S—location of the residues: aspartic acid, histidine, and serine forming the catalytic triad.

Extracellular proteins are located on the cell surface. They are bound to the structure of the cell wall through covalent (homeopolar) bonds [59]. They are synthesized as pre-proteins of approximately 2000 amino acids. Extracellular proteins comprise several domains with different functions [60]. In proteinases bound to the cell wall of LAB, several functional regions can be identified (Figure 2) that are responsible for the complete activation of enzyme precursors (proenzymes).

The PrtM protein is required for PrtP activity in *Lc. lactis* [61]. The *prtP* gene encoding proteinase is preceded by divergent transcription of the *prtM* gene encoding lipoprotein, which is located inside the cell membrane. This protein has a molecular weight ranging from 29 to 33 kDa [62,63]. It is lipoprotein that contributes to the removal of the pro sequence consisting of 154 amino acid residues by autoproteolysis [64]. It should be noted that these two genes are present in *Lacticaseibacillus paracasei* [65], while in *Lactobacillus helveticus*, *Lactobacillus delbrueckii* subsp. *bulgaricus*, and *Lc. thermophilus*, no *prtM* gene has been identified in the regions surrounding the proteinase coding gene [60,61,66].

The first domain occurring at the N-terminus of the enzyme molecule is a preprodome (PP) composed of a signal peptide containing 40 residues and a pro sequence containing 150 residues. The pro sequence section is removed by the autocatalysis process involving the PrtM protein located outside the cell membrane [23,67]. The PP domain is followed by a catalytic serine protease (PR) domain of 500 residues. It contains the active center of the enzyme and is formed by aspartic acid, histidine, and serine. Next, there is the insert domain (I) with 150 residues [4], which probably modulates the proteinase substrate specificity (CEP). Bruinenberg et al. [68] showed that the lack of domain I does not play such a significant role in the activity of extracellular enzymes; it does not occur in *Lacticaseibacillus rhamnosus*. The next domain is domain A (400 residues) with an unknown function. Domain A is located after domain B and is composed of 500 amino acid residues. It is probably involved in stabilizing the activity of proteinases. It has a β-sheet structure. Domain B has been found in most extracellular proteinases, but it is not present in *Lc. thermophilus* [4,23].

The next domain is the H domain composed of an α-helical chain of 200 amino acid residues, which, on the one hand, is attached to the cell by anchoring on the cell wall and, on the other hand, is connected to domain B located outside of the cell membrane. Domain H is present only in PrtP (210 amino acids), PrtS (367 amino acids), and PrtH (72 amino acids) [4,61,69]. Extracellular proteins still include a hydrophilic W domain composed of 100 amino acid residues. This domain contains a typical amino acid composition that is rich in Pro-Gly and Ser-Thr and is similar to the cell wall domain of gram-positive bacteria [23]. In PrtP, PrtS, and PrtR, the W domain has a cell wall anchoring domain (AN) behind it. This domain is very clearly present in *L. lactis* cells. The LAB *Lb. helveticus* and *Lb. bulgaricus* do not have an AN domain, and they are linked to the cell wall through the W domain alone [61]. PrtR found in *Lacticaseibacillus rhamnosus* BGT10 is significantly different from the proteinases of other LAB bacteria, as it lacks both I and H domains [4,70,71].

## 6. Peptide Transport System

Peptides formed due to casein degradation by extracellular proteolytic enzymes are transported into the cells through the functioning of three systems: Opp, DtpT, and Dpp [72]. The Opp proteins belong to the superfamily of ABC transporters [73]. They are coded by five genes (*oppD, oppF, oppB, oppC*, and *oppA*) with monocistronic property [74]. Opp consists of two OppD, OppF nucleotide binding molecules, two integral proteins of the OppB and OppC cell membrane, and an OppA protein that binds oligopeptide [75,76]. These are highly secured ATP-binding cassette transporters, from which obtained energy enables transport of casein-derived peptides across the cell membrane [72,77].This is an example of active transport, which requires energy input and involves specialized integral proteins coupling transport with the energy release process. The oligopeptide transport system (Opp) involves ATP, which transports peptides composed of four to at least eight amino acid residues. The Opp system in *Lc. lactis* transports complex peptides containing 5 to 20 or more, and the nature of these peptides significantly affects transport kinetics [78]. Generally, other LAB Opp systems are not widely studied, but it has been shown that the composition of the Opp system in *Lactobacillus delbrueckii* subsp. *bulgaricus* is similar to that described for *Lactococcus*. In addition, the *Lc. lactis* Opp transporter differs from that of *Salmonella enterica* subsp. *enterica* serovar Typhimurium in that the former can carry larger peptides [79]. It should be noted that *Lc. thermophilus* carries three paralogs of OppA protein-encoding genes, each of which is involved in the internalization of the oligopeptide [4].

Other peptide transporters identified in the bacterial strains *Lc. lactis* MG1363 and IL1403 contain a proton motive force (PMF) dipeptide/tripeptide DtpT and an energy-driven ATP system Dpp (formerly called DtpP) [80]. Dpp can transport di-, tri-, and tetrapeptides containing conditionally hydrophobic branched-chain amino acids (BCAAs) and having the highest affinity for tripeptides, while DtpT prefers more hydrophilic di- and tripeptides. Dpp transports hydrophilic di-peptides such as Glu-Val, Gly-Asp, Met-Asp, Ala-Gly, and Pro-Gly [80]. *Lactobacillus helveticus* also has a DtpT-coding gene that is driven by the PMF protein pump, whose transport specificity resembles its lactococcal counterpart [42,81].

Thus, bacterial growth in milk depends on the presence of oligopeptide transport [82]. It is one of the components of the proteolytic system of LAB that allows these bacteria to transport peptides into the cell where they are further hydrolyzed under the influence of oligopeptidases to free amino acids that are a useful source of energy.

## 7. Regulation of the Proteolytic System

The structure and function of bacterial hydrolytic enzymes and the factors controlling their expression have long been of interest to many researchers. As we know, several studies [4,83,84] have investigated the biochemical characterization of proteolytic enzymes, but there is still little information about the mechanism of action, expression regulation, inhibition, and activation of proteolytic enzymes.

Lactic acid bacteria can regulate the expression of their genes in response to environmental conditions [85]. They respond to changes in the availability of nitrogen by regulating the activity of the proteolytic system to ensure proper nitrogen balance in cells [86,87]. The biosynthesis of specific proteolytic enzymes at the cellular level is based on the mechanism of induction in the presence of a suitable substrate. An important condition is that there should be no other digestible source of energy in the medium that will cause nitrogen catabolic repression. Nitrogen catabolic repression consists of inhibiting the synthesis of enzymes needed to cleave the induction substrate by other readily available compounds. The mechanism of induction and nitrogenous catabolic repression occurs in many microorganisms that produce proteolytic enzymes involved in the hydrolysis of macromolecules and microparticles. Hence, the negative control mechanism (nitrogen catabolic repression) plays an important role in the synthesis of a particular proteolytic enzyme, as it can cause complete inhibition of synthesis. It has been suggested that di/tripeptides with hydrophobic residues behave as effector molecules in the transcriptional regulation of the Opp system and therefore affect the entire proteolytic system in *Lc. lactis* [45,79,88]. Technological advances in the field of molecular genetics, gene expression studies, and proteomic studies have revealed that Opp, PepO1, PepN, PepC, and PepF are regulated in the developmental environment of *Lc. lactis* in the absence of free amino acids and peptides [82]. The CodY transcriptional regulator negatively regulates the expression of several components of the proteolytic system in *Lc. lactis* cells, and the inhibitory potency is modulated in an intracellular environment rich in amino acids such as isoleucine, leucine, and valine [89,90]. While regulating the proteolytic system, the CodY repressor acts as a blocking agent that binds to the CodY operator sequence (AATTTTCWGAAAATT) and prevents RNA polymerase from accessing the promoter sites of individual components of the proteolytic system [4,91,92,93]. To sum up, when the intracellular concentration of the amino acids isoleucine, leucine, and valine increases, several of these amino acids attach to the allosteric site of the CodY repressor by changing the conformation of this protein in a such way that it allows it to bind to the operator in the DNA; consequently, this combination blocks the transcription of genes responsible for enzyme synthesis. However, when the amino acid levels of isoleucine, leucine, and valine are low, the repressor protein is inactive and does not bind to the operator region in the DNA.

Like the composition of the medium, physicochemical factors also significantly affect the regulation of the proteolytic system. These factors include pH, temperature, and dissolved oxygen concentration. The optimal pH for bacterial growth does not always coincide with the optimal pH for the biosynthesis of enzymes. The incubation temperature of the culture also significantly influences bacterial growth and biosynthesis of proteolytic enzymes. The expression of PepO1 and PepC can be induced in an environment rich in BCAA amino acids [4,94].

The regulating mechanism of proteolytic systems in the *Lactobacillaceae* family is less known. The concentration of peptides in the culture environment controls the biosynthesis of PrtH and PrtR in *Lactobacillus helveticus* and *Lacticaseibacillus rhamnosus* [43,70]. PrtH action of *Lb. helveticus* CRL 1062 is inhibited in a peptide-rich environment in which a dipeptide consisting of leucine and proline plays an important role in regulating PrtH activity [43]. The addition of peptides to the culture environment also results in a strong reduction in the expression regulation of the elements responsible for transporting Opp and DtpT peptides in *Lactobacillus sanfranciscensis* DSM 20451 bacterial cells, while the expression of pepT was reduced to a lesser extent [74]. Thus, it was shown that peptides with amino acids such as isoleucine, leucine, and valine act as corepressors that deactivate the activity of the CodY repressor.

Expression of most elements of the proteolytic system depends not only on the concentration of amino acids and peptides in the culture medium, but also on the presence of another energy source. It has been shown that a carbon source in a growth environment affects the expression of the pepP enzyme in *Lc. lactis* [95]. PepR1 (prolase) biosynthesis depends on the concentration of glucose in the medium. The expression of prolidase also depends more on the composition of carbohydrates in the medium than on the concentration of peptides, which contrasts with the biosynthesis of other components of the proteolytic system in *Lactobacillus delbrueckii* subsp. *bulgaricus* [96,97].

## 8. Industrial Use of Proteolytic Enzymes

**Figure 3:**
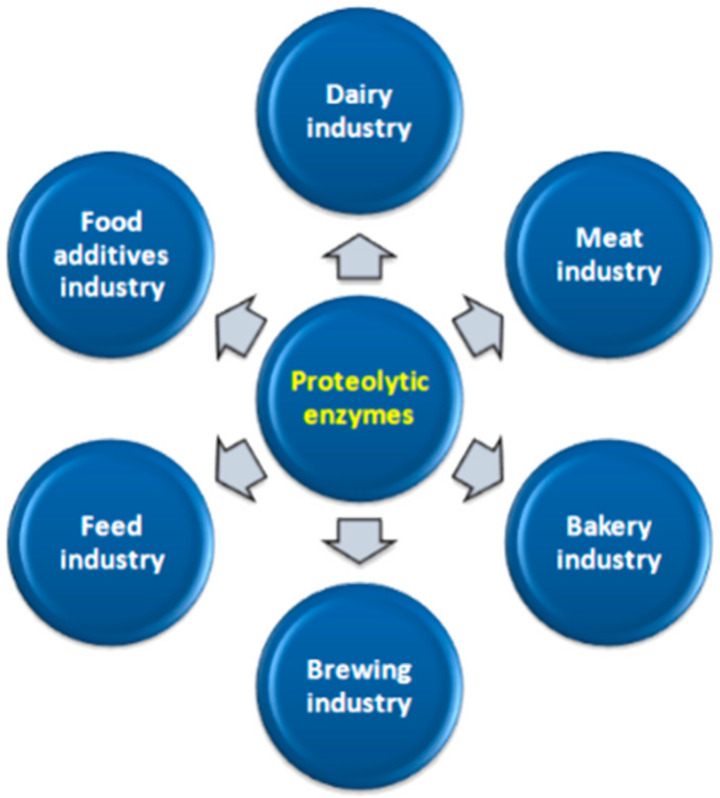
Examples of industrial use of proteolytic enzymes.

Proteolytic enzymes of bacteria and fungi are an extremely important group of enzymes used in industrial processes (Figure 3). They are produced by gram-negative bacteria, and gram-positive bacteria. The diverse catalytic abilities of proteolytic enzymes enable their widespread use in various industries. Today, the greatest expectations for the practical use of proteolytic enzymes are related to the possibility of using them in the dairy industry. In the dairy industry, LAB play an important technological role in creating the rheological and sensory properties of the obtained products.

### 8.1. Dairy Industry

In the dairy industry, microbial cultures characterized by a rich enzymatic array, especially with proteolytic activity, are widely used in the production of cheese, yogurt, kefir, and other so-called fermented dairy products. As is known, proteolytic enzymes are used not only to hydrolyze proteins but also to coagulate milk proteins in the cheese-making process. Various protein hydrolysates are obtained from milk using proteolytic enzymes. Easily absorbable dairy products are produced for sick people and children. The exogenous proteolytic enzymes used for cheese production are very important [6,98,99,100,101]. Some microbial proteases produced by LAB may replace chymosin in cheese production [102]. *E. feacalis* is able to produce active protease, which indicates the possibility of its application to support the breakdown and release of bioactive peptides from whey protein [99]. LAB (*E. faecalis* VB43) also produce enzymes that are capable of hydrolysing allergenic proteins in milk, so they have good potential for the manufacture of hypoallergenic dairy products [103].

### 8.2. Meat Industry

Under the influence of proteases in meat, partial macromolecules of muscle proteins; disruption of disulfide bonds; and increases in the reactivity of sulfhydryl, hydroxyl, carboxyl, and amino groups occur. During this process, sarcoplasmic proteins are hydrolyzed to a significant extent, and depending on the specificity of the enzymes, myofibrillar and connective tissue proteins are partly hydrolyzed. The meat becomes tender, the degree of its hydration increases, and the protein digestibility increases; i.e., more meat is available for proteolytic digestive enzymes. Because of these processes, the content of soluble and free amino acids and small peptides increases, which improves the palatability of meat. Fragility also improves, and its maturing time is shortened. Proteolytic enzymes contribute to the improvement in taste and are a protective factor against many unfavorable microorganisms. They affect the proper meat texture, aroma, and color, and an even better sausage cut [104,105,106,107,108]. The addition of proteases to meat may delay lipid oxidation [109].

### 8.3. Bakery Industry

In this case, proteolytic enzymes are used in the production of bread and pastry. Enzymes of LAB contribute to the degradation of gluten protein, thus affecting the rheology of sour wheat dough and ultimately the texture of bread [110].

*Lactobacillus sanfranciscensis* is an interesting species in the baking industry, especially for the production of wheat-based cakes. This is because of the unique features of this strain, expressed in terms of high efficiency of saccharide use, high proteolytic ability and acidifying activity, synthesis of antimicrobial compounds, production of a wide range of volatile compounds, and physiological affinity for the yeast present in sourdough [111,112].

The properties of bread, its quality, and flexibility depend on the state of the proteins it contains. The protein content in flour ranges from 6% to 20% by weight. As is known, flour contains proteolytic enzymes; however, their amount is insufficient. Hence, a certain amount of enzyme needs to be added for the beneficial hydrolysis of protein substances. The addition of proteolytic preparations is also used in the production of confectionery. Such preparations are also successfully used for the production of cereal grain products. The action of enzymes shortens kneading time, softens the dough, and increases its volume. Proteolytic enzymes are particularly important in the baking and pasta industries because of their strong effect on the rheological properties of the dough and, consequently, on the quality of the finished product. LAB and their proteolytic enzymes in sourdough contribute to the improvement of the dough baking process, improve the taste and aroma of the bread, extend its shelf life, and contribute to the improvement of yeast’s dough raising capacity [33,113,114,115,116].

### 8.4. Brewing Industry

In the brewing industry, proteinases are used to dissolve proteins and prevent beer from becoming cloudy when it is cooled. It is worth mentioning here that the activity of native proteases is observed in the process of grain malting (usually barley); they contribute to clarifying the wort and raising its value, both as a substrate for yeast and as a final food product. It should be noted that a uniform protein distribution is not desirable, as the foamy beer is reduced and becomes “empty” in taste. On the other hand, insufficient protein distribution makes filtering more difficult and causes beer to become cloudy during storage [33,117,118,119,120,121,122].

### 8.5. Food Additives Industry

Proteases can be used for the removal of the cell mass in the production xanthan gum of *Xanthomonas campestris* [102]. Proteolytic enzymes are used to produce food concentrates, protein hydrolysates, and soy sauces [102,123,124].

Many lactic acid bacteria has proven probiotic-synthetic activity, thereby contributing to the health of consumers. Probiotics are live microorganisms that occur in a certain amount in food, which, when consumed, benefit the health of the host by improving its intestinal microflora. The use of such bacteria as food additives may be an ideal factor modulating the proteolytic activity of a given food product [125].

### 8.6. Feed Industry

Keratinolytic peptidase could be used for the degradation of fibrous animal protein (keratin) from feathers, horns, hair, and nails available as natural waste. The products obtained in this way could supplement animal feed [105]. Proteolytic enzymes can be used in the processing of eggs into highly nutritious animal feed [126]. Proteolytic enzymes showed non-specificity in the amino acid studies evaluated in various studies leading to improved digestibility of animal feed [105]. In summary, it can be stated that the pursuit of high profitability in animal production has resulted in the introduction of various preparations in animal nutrition on a large scale. The attractiveness of LAB due to their proteolytic properties and high probiotic activity suggests that such preparations will be increasingly used for industrial, agricultural, preventive, and even therapeutic purposes. Thus, the use of pro-ecological technologies that use the enzymatic properties of bacteria is one of the most important areas of activity in obtaining safe food [72].

## 9. Conclusions

Proteolytic enzymes are a diverse group of enzymes that catalyze several different biochemical reactions. A growing interest in these enzymes stems from the possibility of their use in many industries. Research on proteolytic enzymes is focused on understanding the optimal operating conditions and determining the biochemical properties, specificity, and primary structure of these enzymes that will allow the maximum use of their properties in various industries. The complete control of the proteolysis process can ensure the yield of the highest quality products; it can also improve technological processes and further reduce production costs. However, this requires thorough knowledge of these enzymes: knowledge of the variations in proteolytic system components may facilitate the anticipation of the proteolytic and flavor-forming potential of LAB bacterial strains. This information could be applied, e.g., to improve the aesthetic characteristics of dairy and other fermented food products by supporting the strain selection process.

The latest advances concerning LAB, their genetics and microbiology, may identify proteolytic enzymes whose properties are close to industrial requirements. The increasing number of genome sequences of lactic acid bacteria species allows the integration of functional genomic methods (transcriptomics, proteomics, and metabolomics) to clarify the character of metabolic pathways of lactic acid bacteria and their regulatory mechanisms. It is also possible to manipulate the proteolytic system to produce bioactive peptides of particular interest. Together with novel food-grade genetic tools, this knowledge will support the screening of new LAB strains that will perform better in the fermentation processes to deliver healthy food with an appropriate flavor, smell and texture. What is more, the production of enzymes is also currently experiencing an impressive development. In the future, proteolytic enzymes may have many new applications, not only in food technology, but also in the pharmaceutical and chemistry industries.
